# Current Status and Future Trend of Dominant Commercial Reverse Osmosis Membranes

**DOI:** 10.3390/membranes11110906

**Published:** 2021-11-22

**Authors:** Masaru Kurihara

**Affiliations:** Toray Industries, Inc., 3-2-1 Sonoyama, Otsu 520-0842, Japan; masaru.kurihara.z9@mail.toray

**Keywords:** desalination, distillation, reverse osmosis, cross-linked fully aromatic polyamide composite membrane, forward osmosis, carbon nanotube, pore size and morphology analysis of protuberance, low-pressure SWRO, solar energy, green desalination

## Abstract

Since 2000, seawater reverse osmosis method has been a dominant desalination technology against the distillation method in the global market. The large project called “Mega-SWRO” (half mega-ton per day and larger) plant in the Middle East is quite popular making full use of the combination with solar energy. Today, the price of desalinated water is affordable at as low as $0.28/m^3^ to $0.53/m^3^. Likewise, dominant commercial reverse osmosis membrane is a cross-linked fully aromatic polyamide composite membrane-spiral wound element including FT-30 (DuPont Water Solution) and UTC-80 (Toray Industries., Inc., Otsu, Shiga, Japan). The said membranes are much superior in terms of performance compared to the cellulose triacetate membranes-hollow fiber for variety of applications including seawater desalinations, brackish water desalination, wastewater reuse, ultra-pure production for semiconductor, home-use water purifier, etc. SWCC of Saudi Arabia has announced that it intends to shift from cellulose triacetate hollow fiber to spiral wound RO membranes at all of its plants. Furthermore, the state-sponsored R&D on membrane and membrane process has been put into practice in major countries, including Japan and Korea, which contributed to the progress of membrane science and membrane process, suitable for spiral-wound polyamide membranes. SWCC has announced their plans for SWRO, mainly focusing on brine mining to obtain precious materials from the brine of SWRO. New and innovative brine-mining technology has been introduced for green desalination.

## 1. Introduction

### 1.1. President Kennedy’s Speech

President Kennedy delivered a speech (1961) at a news conference on a desalination plan to “see the deserts bloom” on 12 April 1961 [1]. His desalination plan was as follows:

If we could ever competitively, at a cheap rate, get fresh water from salt water, that it would be in the long-range interests of humanity which could really dwarf any other scientific accomplishments. I am hopeful that we will intensify our efforts in that area.

President Kennedy gave this speech 17 times during his career in the Senate, nine times before he was appointed to the presidency, and eight times after taking office.

### 1.2. Tambo’s Prediction

**Figure 1:**
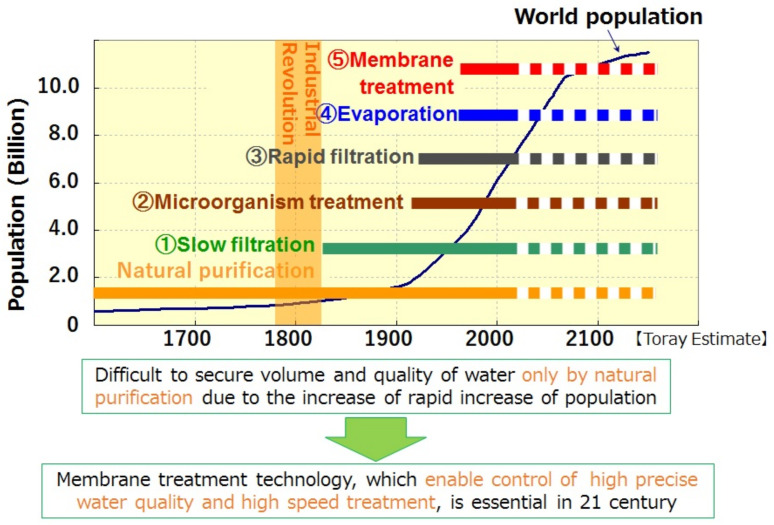
Increase of world population and development of water treatment technologies [2].

Tambo’s prediction (Tambo, 2002) on the increase in the world population and the development of water treatment technologies are shown in Figure 1 [2]. From Figure 1, evaporation (Distillation) and membrane treatment is the latest technology among other conventional technologies on a quite long period of time. Membrane treatment technology, which enables highly precise control of water quality and high- speed treatment, is an essential countermeasure to the water shortages we face in 21st the century.

## 2. Discussion

### 2.1. Current Global Market Status of SWRO Desalination Plants

#### 2.1.1. Technology Transition from Distillation to Membrane Method

**Figure 2:**
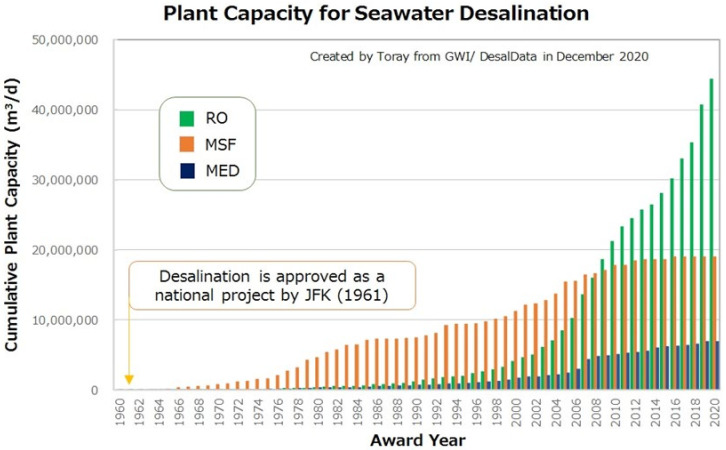
Technology transition of distillation to membrane [3,4].

The United States undertook a research and development on seawater desalination systems including the distillation process and membrane process in the early 1960s. The distillation process became a major technology used in actual plants in the 1970s. Around 2010, there has been a technology transition from the distillation to the reverse osmosis membrane process, as shown in Figure 2. Today, the membrane process has become a major technology (Global Water Intelligence: Desal Data, Desalination Projects, December 2020) [3,4].

The cumulative online capacity (m^3^/day) of RO is much higher than that of MSF and MED. Likewise, the growth rate of RO is also much higher than MSF and MED. The large number of RO plants implies that the average size of RO plants will still be smaller compared with MSF and MED.

However, since 2018, actions have been taken towards the development of “Mega-SWRO” in the Middle Eastern countries. Considering the dramatic change from distillation to SWRO in those countries, the desalination market forecast for the ratio of the Gulf and the rest of the world falls between 45 to 55%. 2018 and 2019 were important years for SWRO. More than 6 million m^3^/d (1585 MGD) of new production capacity have been contracted during that time.

#### 2.1.2. From Small Plants to “Mega-size SWRO” Plants

**Figure 3:**
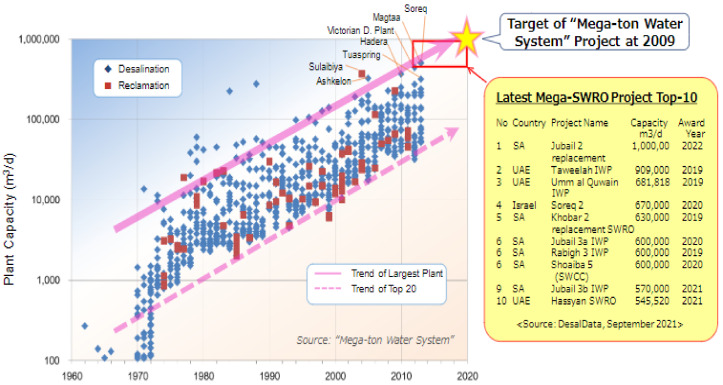
Global SWRO desalination plant capacity development [5,6,7].

The trend of plant capacity and the 20 largest RO plants since 1970 are plotted in the left side of the Figure 3 based on the report made in 2009 [5]. The scale of each desalination plant has been increasing year by year, thus we predicted in 2009 that the market will be in need of “Mega-SWRO”: large plants of the mega-ton per day scale (1,000,000 m^3^/day) by 2020. This prediction has turned out to be right as shown on the right of Figure 3 [5,6,7]. Construction started on many large plants over the 500,000 m^3^/day, so-called ”Mega-SWRO,” between 2018 and 2020 in the Middle Eastern countries such as Saudi Arabia and the UAE. 2018/21 Tenders are shown on the right of Figure 3, presented at the Saudi Water Forum in 2019 by Mr. Altmann [6] and by the authors [7].

#### 2.1.3. Affordable Price of Desalinated Water

**Figure 4:**
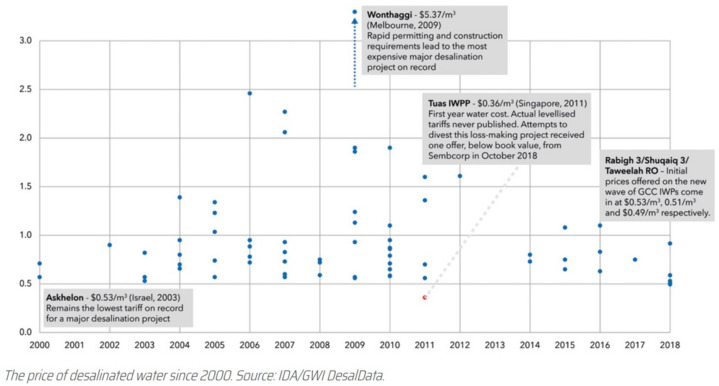
Rapid change of the desalinated water price since 2000 [7].

The rapid changes in the price of desalinated water since 2000 have been presented by Mr. Christopher A Gasson as shown in Figure 4 [7].

The price of desalinated water in large-size plants (Mega-SWRO) has rapidly decreased since 2018 [7].

Rabihg 3 (Saudi Arabia): 600,000 m^3^/d, $0.53/m^3^
Shuqaiq 3 (Saudi Arabia): 380,000 m^3^/d, $0.51/m^3^
Taweelah (UAE): 909,200 m^3^/d, $0.49/m^3^
Jubail 3A (Saudi Arabia): 600,000 m^3^/d, $0.41/m^3^
Soreq 2 (Israel): 672,000 m^3^/day, $0.40/m^3^
Hassyan (UAE) (Saudi Arabia): 545,000 m^3^/d, $0.28/m^3^
The price has dropped to $0.28/m^3^ in December 2020.

### 2.2. History of RO Membrane Materials Configuration: Hollow Fiber, Spiral Wound Element, and Module

#### 2.2.1. History of Membrane Materials and Configuration

In the early 60s, it was shown that Loeb-Sourirajan cellulose acetate membrane was asymmetric, having the thin dense layer over a microporous film. Asymmetric membranes resulted from casting a single polymer solution in one step, composite membranes, on the other hand, are formed in two steps, casting of microporous support first, followed by deposition of barrier layer on the surface of this microporous support layer [8,9,10,11].

Typical commercial examples of asymmetric membranes were cellulose acetate-spiral wound element by U.O.P. and linear fully aromatic polyamide-hollow fiber membrane by Du Pont in the late 60s as shown in Table 1 [12]. Although the early stage of research on the composite membrane using cellulose acetate has started at North Star Research Institute and U.O.P. in the U.S. in the middle of the 60s, the authentic and severe competition of R&D on composite membrane as post asymmetric membranes has been done between the U.S.A. (North Star Research Institute and U.O.P.) and Japan (Toray and Teijin) [9,10].

Since the late 70s, rapid progress has been made in the development of commercial composite membranes. A big and memorable conference was held in Nice, France, 1979 by 4th International Desalination and Environment Association (IDEA). During the conference, four companies from the U.S. and Japan made presentations on seawater desalination membranes including Du Pont (B-10: linear aromatic polyamide-hollow fiber membrane/asymmetric type), Film Tech (FT-30: Cross-linked fully aromatic polyamide-spiral wound composite membrane), Toyobo (CTA hollow fiber membrane-asymmetric type) and Toray (PEC-1000 spiral wound composite membrane) as shown in Table 1.

FT-30 is prepared by the interfacial polymerization of aromatic diamine and aromatic triacyl halide on the surface of the support film. Meanwhile, PEC-1000 membrane is prepared by an acid catalyzed in siti poycondensation reaction of 1.3.5.-tris (hydroxyethyl) isocyanuric acid and furfuryl alcohol on a microporous support.

The main discussion emphasized the membrane performances and membrane durability:

Seawater desalination process: One-stage or two-stage.
Water recovery: 10–20% or 30–40%
Chemical stability of the membrane against chlorine and dissolved oxygen in seawater.
Physical stability of the membranes to the temperature of seawater.

PEC-1000 composite membrane showed one-stage seawater desalination process at 40% water recovery, however, Toray switched from PEC-1000 composite membrane to UTC-80 composite membrane in 1987 due to the lack of dissolved oxygen resistibility in PEC-1000. Toray’s UTC-80 composite membrane is prepared by the interfacial polymerization of the aromatic diamine and triamine with aromatic diacyl halide and triacyl halide on the microporous support film as shown in Table 1.

Before Du Pont (before the merger with Dow) had closed their membrane business related to B-10 in 1999, it also developed A-15 membrane. A-15 membrane is the cross-linked aromatic/alicyclic polyamide composite membrane-spiral wound element. As shown in Table 1, this membrane has not been commercialized by Du Pont themselves.

#### 2.2.2. The Global Market Share of Spiral-Wound Element and Hollow Fiber Type Element

In 2020, a commercial reverse osmosis membrane in 2020 “cross-linked fully aromatic polyamide composite membrane-spiral wound element” was prepared by the interfacial polymerization of aromatic amines and aromatic acyl halides such as FT-30 by Du Pont Water Solution (formerly Dow Chemical) and UTC-80 by Toray Industries, Inc. Other suppliers of cross-linked fully aromatic polyamide composite membrane-spiral wound element are Hydranautics, and LG Chemical. On the other hand, Toyobo is the only supplier of asymmetric CTA hollow fiber.

**Figure 5:**
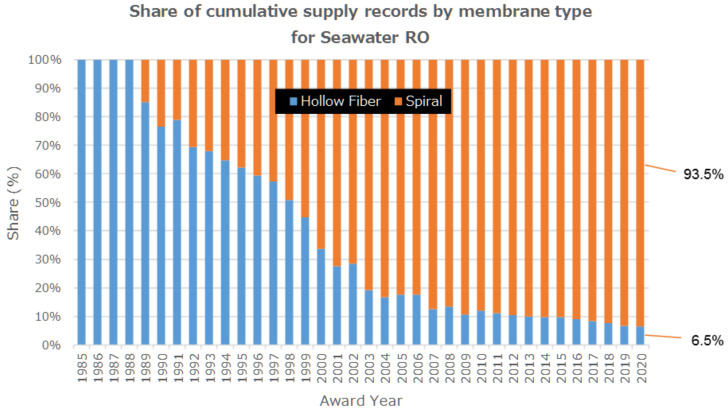
Share of cumulative supply record by membrane configuration type [13].

The global market share of cross-linked fully aromatic polyamide composite membrane-spiral wound element to the hollow filter type reached 93.5% in 2020 as shown in Figure 5 and 6.5% for hollow fiber type [13].

**Figure 6:**
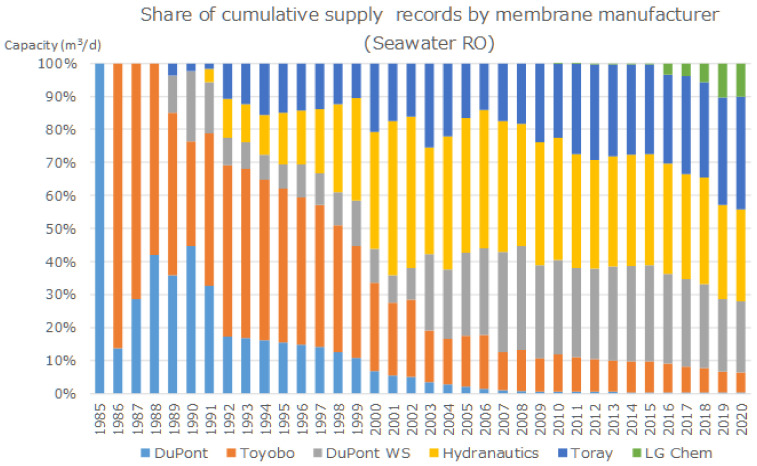
Share of cumulative supply records by membrane manufacturer [13].

Figure 6 shows the share of cumulative supply records by membrane manufacturer (seawater RO).

As shown in Figure 5, hollow-fiber type elements were manufactured by DuPont (linear aromatic polyamide membrane) and Toyobo (cellulose triacetate membrane), however, after 2006, Toyobo started to monopolize the market share [13]. On the other hand, there are four key spiral-wound membrane manufacturers for seawater desalination: Du Pont Water Solution, Toray, Hydranautics, and LG Chem. Figure 5 shows that only spiral –wound element is used in SWRO after 2010.

#### 2.2.3. SWCC’s Vision for the Future

H. E. Eng. Abullah Bin Ibrahim Al-Abddulakareem, Governor of Saline Water Conversion Corporation (SWCC) presented “SWCC’s vision for future: three initiatives” in Global Connections Spring 2021 as highlighted below [14]:

(1) Replacement of their thermal desalination plants with state-of-the-art RO plants and enhancement of exiting plant operations.

(2) Switching from hollow-fibre to spiral wound RO membranes at all of their plants.

(3) Developing new generation RO membranes, energy recovery devices and pump systems of enhanced energy efficiency and adopting green chemical and brine mining initiatives.

The item (2) corresponds to Figure 5 and Figure 6. SWCC’s vision for the future showed no more use of CTA-hollow fiber membrane in SWCC plants. Therefore, the membranes used in the latest Mega-SWRO project top 10 shown in Figure 3 are “cross-linked fully aromatic polyamide composite membrane-spiral wound element. Four major suppliers of spiral-wound element divided SWRO plant market.

### 2.3. The Pursuits of Existing Ultimate Membrane Technology and Membrane Process

#### 2.3.1. Trend of Global Membrane Research on SWRO Membranes

Currently, there are four major manufacturers supplying commercial SWRO membrane elements based on cross-linked fully aromatic polyamide composite membrane spiral wound elements: Toray Industries (Japan), DuPont Water Solution (U.S.A.), Hydranautics (U.S.A.), and LG Chemicals (Korea). Although SWRO membranes are constantly evolving due to an advancement made by key membrane manufactures, there has not been much report made on the progress of SWRO membrane development.

Since 2000, the market of desalination started to grow rapidly in SWRO, the targets of membrane technology have become very clear, that is to reduce energy consumption, environment load, and water production costs [7,15,16].

**Figure 7:**
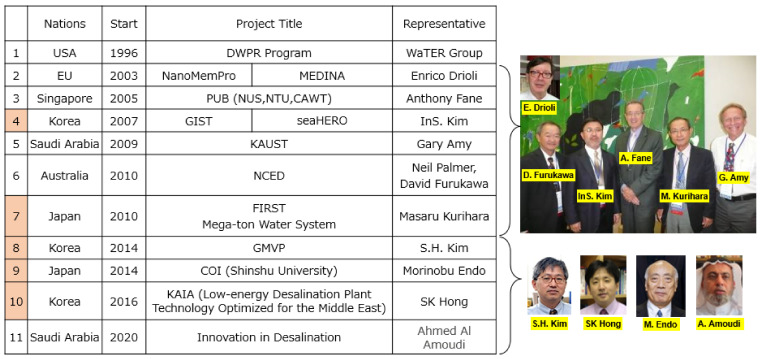
State-sponsored R&D on membrane and membrane process [15].

State-sponsored R&D on membrane and membrane process became very popular in the main global countries as shown in Figure 7 [15].

As shown in Figure 7, Korea had three projects during 2007–2016. A project implemented by Prof. InS. Kim had been pointed to SWRO. Prof. S.H. Kim carried out membrane distillation method and other future essential membrane technologies. Project implemented by Prof. SK. Hong was associated with low-energy desalination plant technology optimized for the Middle East by combining it with FO-SWRO, so-called pressure-assisted forward osmosis (PAFO)-reverse osmosis (RO) hybrid process. The same concept was also presented by InS. Kim at IDA World Congress held in Dubai from 20 to 24, October 2019 [16,17].

In the meantime, two national projects had been implemented in Japan: Mega-ton Water System by Kurihara and COI by Prof. Endo [18,19].

One of the examples of state-sponsored R&D on membrane and membrane process was “Mega-ton water system” project in Japan, implemented during 2009–2013 by Kurihara [5,7,15,20]. This project clarified the precise structure of pore size and protuberance in the functional layer of composite polyamide membrane on a scientifically basis. As a result, by using these basic scientific analysis, high performance SWRO membrane so-called low pressure SWRO membrane has been obtained, which contributes to the reduction of the energy consumptions described in the following chapters.

#### 2.3.2. Progress of SWRO Membrane Technology in “Mega-Ton Water System” Project

(1) Membrane structure analysis of polyamide layer.

Requirement of water quality improvement.

**Figure 8:**
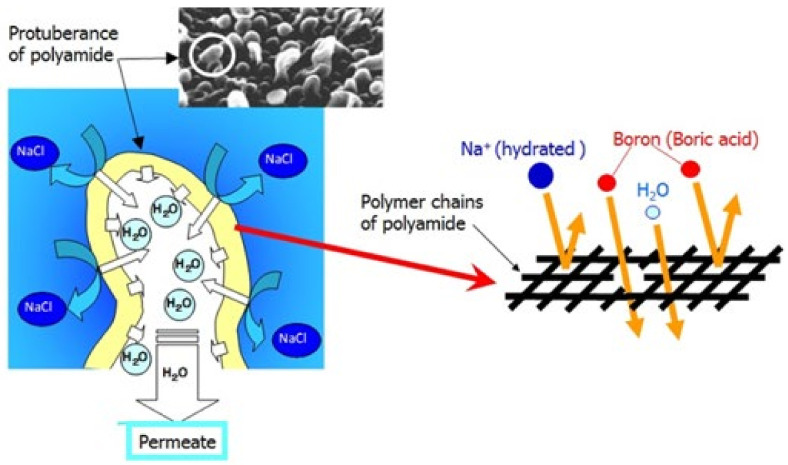
Schematic diagram of salt removal and water permeation through the protuberance [15,20,21].

Although the ideal cross-linked fully aromatic polyamide SWRO membrane should have both high water permeability and high solute removal performance, generally, there is a trade-off between an increased water permeability and decreased solute rejection rate. However, the performance of an RO membrane is controlled by altering the size and quantity of the pores in the membrane, which are spaces within polymers. Namely, solutes in water are excluded by pore size, and the water permeability depends on the quantity of pores. To enhance performance, it is necessary to conduct a scientific research on the molecular structure and solute transport mechanism in an RO membrane. A schematic diagram of water permeation through the protuberance of the RO membrane is shown in Figure 8 [15,20,21].

**Figure 9:**
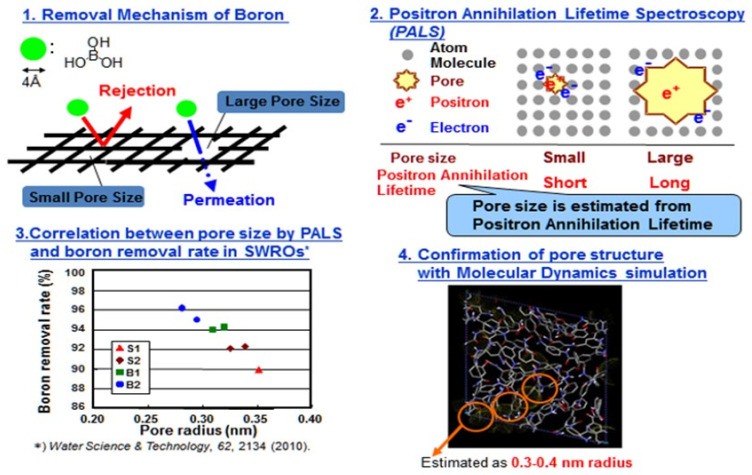
Pore size estimation of RO membrane [15].

Pore size analysis for separating functional layer in polyamide composite membranes has been conducted based on Position Annihilation Lifetime Spectroscopy (PALS) study [15] and it showed the pore sizes in the range of 5.6 to 7.0 Å (see 3 in Figure 9). It was considered that this range of pore in the separating layer would characterize the membrane property. Furthermore, the correlation between pore size of RO membrane and boron permeability was revealed as shown in 3 in Figure 9.

The molecular dynamics simulation results showed that the pore sizes were estimated to be 6 to 8Å (see 4 in Figure 9).

(2) Progress of RO membrane performance

On the basis of this knowledge, Toray has developed new RO membrane elements with high solute rejection performance for SWRO processes. The lineup of RO membrane elements for SWRO processes is shown in Table 2 [20].

TM820C shows 93% of the boron rejection rate with high total dissolved solids (TDS) rejection rate. TM820E and TM820M have both high boron rejection rate and high water productivity. TM720C is utilized for second stage in multistage process due to the tolerance of alkaline agent. And most recently, TM820R, which has achieved coexistence of high TDS and boron rejection rate and high water productivity, has been released. TM820R has already been run with high performance and stable operation.

To develop innovative low-pressure SWRO membranes aiming at energy reduction, it is necessary to evaluate the pore size of the RO membrane functional layer and the fold.

**Figure 10:**
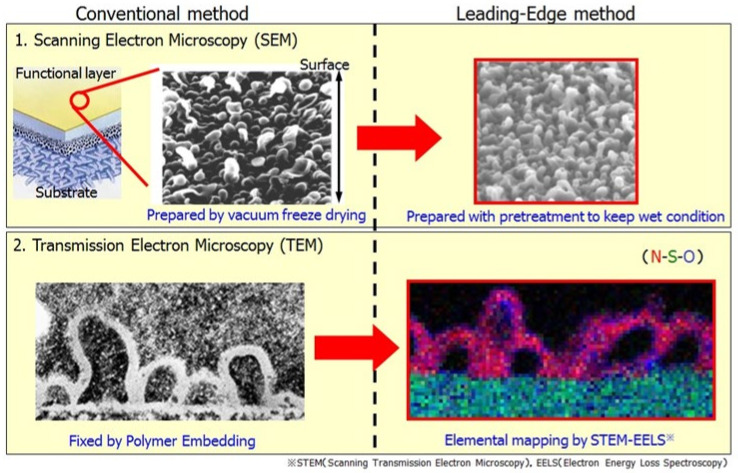
Precise structure of protuberance in “Mega-ton Water System” project [15,20,21].

Using a conventional scanning electron microscope (SEM), only the information shown in Figure 10 (upper left) was obtained, and the fold structure could not be determined accurately. In this research, an improvement was made to the conventional pretreatment method of the RO membrane structure analysis, and a new pretreatment method that can be analyzed and evaluated while maintaining the membrane surface morphology in the wet state of the membrane was developed. As a result, it has become possible to capture the fold structure accurately as shown in Figure 10 (upper right) [15,20,21].

By applying the new pretreatment method and adding technology for evaluating basic analysis of the RO membrane structure using a transmission electron microscope (TEM) (conventional method (Figure 10) (lower left)), it has become possible to perform mapping analysis on nitrogen atoms derived from polyamide membranes. This has made it possible to clarify that the surface of the fold structure is a true polyamide layer (Figure 10) (lower right) [15,20,21].

Scanning transmission electron microscope with electron energy loss spectroscopy (STEM-EELS) characterization revealed that the skin of the protuberances is the real polyamide layer (~200 nm in height) that consist of a core polyamide layer (~20 nm in thickness) [15,20,21]. It must be highlighted that there exist a difference between the polyamide height and thickness, whereby the former depicts the visual height consisting of the protuberances (~200 nm) and the latter represents the core separation layer (~20 nm) embedded within the PA ridge and valley structure [15,20,21].

Since the RO membrane structure analysis technology described above made it possible to specify the factors of an ultrathin membrane fold structure related to the RO membrane performance, to obtain guidelines for the design of an RO membrane whose fold structure was captured in three dimensions as well as obtain an innovative low-pressure SWRO membrane capable of energy saving, it was found that the optimum pore size control of the RO membrane functional layer and optimization of the surface area and membrane thickness of the folds were important.

(3) Innovative low-pressure SWRO membrane

**Figure 11:**
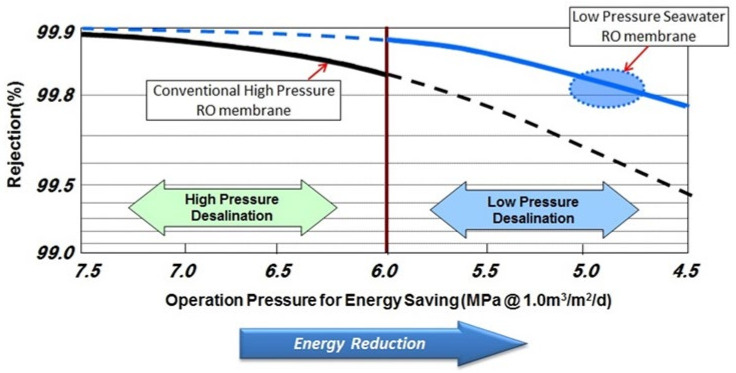
Comparison of conventional high-pressure Seawater Reverse Osmosis (SWRO) membrane and low-pressure SWRO membrane [15,20,21].

As a result, basic membrane forming technology on a bench scale was established, and the formation of an energy reduction type low pressure SWRO membrane that can operate at a significantly lower pressure compared to the conventional RO membrane turned out to be a success (Figure 11) [15,20,21].

**Figure 12:**
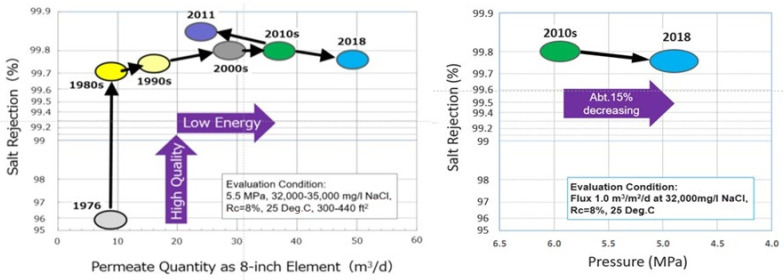
History of the high-performance SWRO membrane: Toray “ROMEMBRA” SWRO Membrane and new advanced low pressure SWRO membrane [15,20].

The operation pressure to obtain the same permeation flux and the same salt removal rate is higher than 6 MPa in conventional high-pressure desalination. However, the low-pressure desalination in this research makes it possible to lower the pressure to 5 MPa, greatly contributing to energy saving (Figure 12).

The history of the high-performance SWRO membrane and the innovative low-pressure SWRO membrane developed by Toray are shown in Figure 12 [15,20]. Since the 1980s, the membrane performance of cross-linked fully aromatic polyamide composite membranes has been continuously improved.

Both in salt rejection (%) and permeate quantity (m^3^/d as 8-inch element) as shown on the left side of Figure 12 [15,20]. The right side of Figure 12 [15,20] shows a comparison at the same flux base between the latest innovative low-pressure SWRO membrane (2018) and the 2010s membrane. About 15% decrease of operating pressure was confirmed as an advantage of the low-pressure SWRO membrane.

(4) Low-pressure, two-stage, high-recovery SWRO system

In the development of RO technology, continuous research is required on membranes and membrane processes.

(I) Brine conversion two-stage, high-recovery SWRO system.

**Figure 13:**
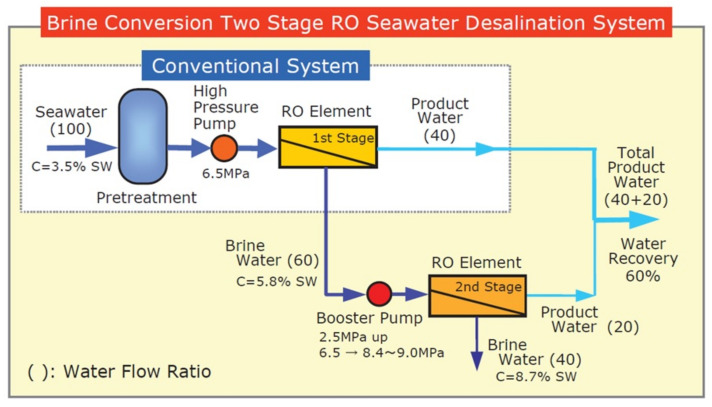
Brine conversion two-stage high recovery system (BCS) [22].

A low-cost seawater desalination system called Brine Conversion System (BCS) was developed 20 years ago by the Toray Group [22], as shown in Figure 13, which provides a 60% recovery rate. The concentrated seawater (5.8% of salt concentration) from the first stage RO modules is pressurized to 8–10 MPa by a pressure booster, then the concentrate is supplied to the second stage RO modules. Additional fresh water is obtained from the second stage, and the feed water is finally concentrated to approximately 8.7% salt concentration. A 60% recovery rate in total can be obtained, of which 40% comes from the first stage and a further 20% comes from the second stage.

To achieve 60% recovery in a RO seawater desalination system, the RO membrane element must be durable under very severe operating conditions with 10 MPa of high pressure and 5.8% of high feed water concentration. Toray developed a high-pressure durable BCS element.

The advantages of the BCS system compared with the conventional system are as follows:

a. Plant installation space can be reduced by 1/3.

b. Plant capacity is easily expanded 1.5 times by only adding BCS second stage into a conventional plant.

c. Disposed concentrated brine water is reduced by 1/3.

Therefore, the BCS is effective in reducing the footprint, power and cost of a SWRO desalination plant. The BCS system has been operated in actual plants, for example, in Trinidad and Tobago (136,000 m^3^/day) and in the Canary Islands (14,000 m^3^/day) for over 15 years. This two-stage SWRO system is still an important research theme of the SWRO membrane process [23,24].

(II) Low-pressure two-stage high-recovery SWRO system (LMS)

The “Mega-ton Water System” research project uses the world’s first high-performance innovative low-pressure SWRO membrane described in Section 2.3.2 (2) as a basic element technology, and Hitachi developed the LMS to prevent fouling of the RO membranes and to enable RO elements to be utilized effectively in order to enhance the reliability of plant operation [25,26]. This system makes it possible to greatly reduce the equipment cost, operation cost, and energy required by enlarging the equipment parts and system and downsizing the water intake/pretreatment facilities (30% reduction of the installation area) by enhancing the recovery rate.

**Figure 14:**
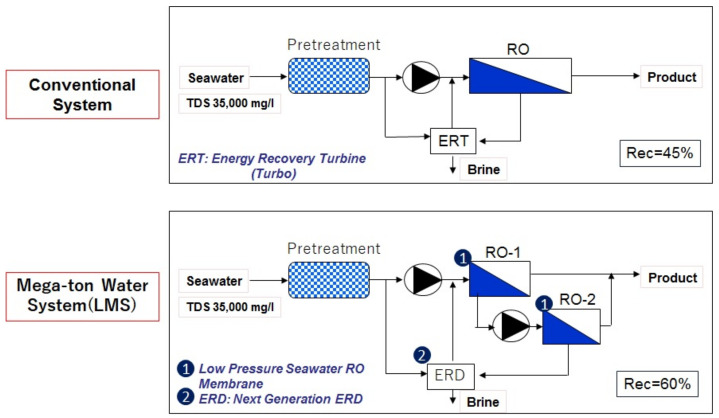
Comparison of flow diagram for conventional Seawater Reverse Osmosis (SWRO) system and the “Mega-ton Water System” (Low-Pressure, Multi-Stage System (LMS)) [25,26,27].

The LMS incorporating low-pressure SWRO membranes and a new Energy Recovery Devise (ERD) [27] under high recovery rate, which will reduce the facility costs and the supply of seawater, is a system that derives the maximum performance of the RO membranes. Figure 14 shows a comparison between the conventional system flow with a 45% recovery rate and the megaton system (LMS) flow with a 60% recovery rate [25,26,27].

(III) What energy saving can achieve with and without advanced energy recovery system.

## 3. SEC Rate Comparison of Conventional SWRO and “Mega-Ton Water System” (LMS)

**Figure 15:**
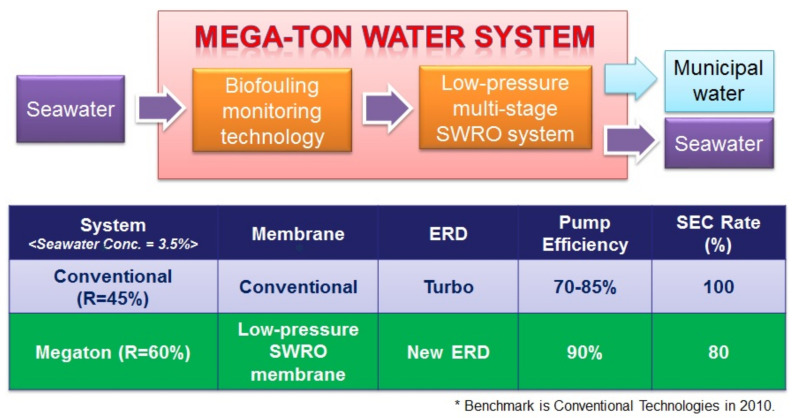
SEC Rate (%) comparison of conventional SWRO and Mega-ton System (LMS) [25,26,27].

The “Mega-ton Water System”, combining the research results described above with the latest high-efficiency, high-pressure pump technology (manufactured by Ebara Corporation), made it possible to dramatically reduce energy consumption [26,27]. It also enabled the energy savings of 20% compared to conventional seawater desalination systems technology from 2010 using a turbocharger-type ERD, as shown in Figure 15 [25,26,27].

Although Miguel Sanz has reported the historical trends of the energy consumption of the total plant and the 1st RO pass. In the “Mega-ton Water System” project, the target value of energy consumption as a total plant under TDS 35,000 mg/L seawater concentration was set to 2.80 kWh/m^3^ or less. In this case, it is possible to reduce energy by 20% compared with the conventional system [25,26,27].

**Figure 16:**
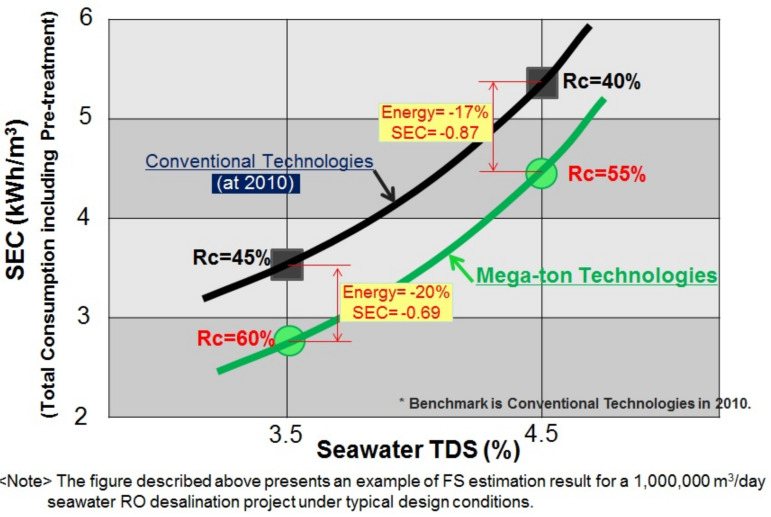
The relationship between specific energy consumption (kwh/m^3^) and seawater TDS (%) [28,29,30].

Technologies of the “Mega-ton Water System” will contribute to large energy reductions for large-scale desalination plants even under high salinity seawater conditions as shown in Figure 16. This also exhibits the relationship between SEC and seawater TDS as a comparison of conventional technology and “Mega-ton Water System” technologies.

20% energy reduction will be expected in the case of “Mega-ton Water System” technology for 3.5% TDS and 17% for 4.5% TDS [28,29,30].

**Figure 17:**
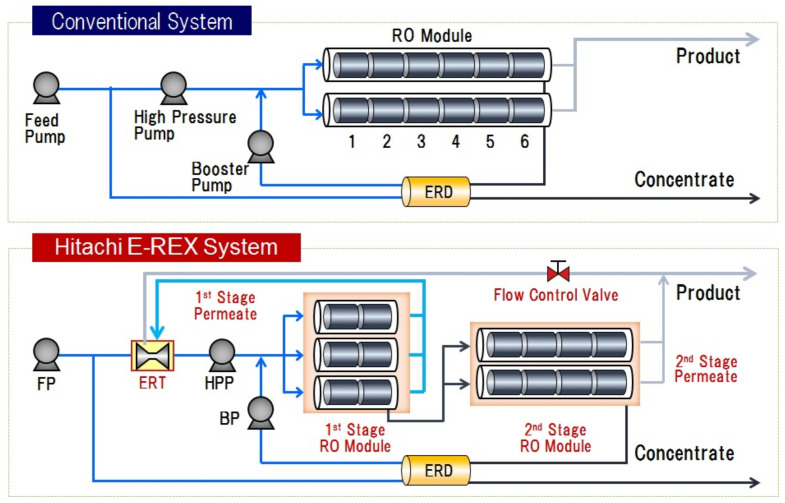
High recovery—Low fouling RO design configuration [31].

As shown in Figure 17, a high-recovery and low fouling RO design configuration have been also announced as the key features of Hitachi E-REX system (developed by Hitachi) [31].

### 3.1. Low Environmental Impact and Reliable Plant Operation: Green Desalination

#### 3.1.1. Green Desalination

In the last 13 years, there has been an increasing awareness of the need of environmental preservation and various efforts have been made to reduce the amount of chemically treated seawater discharged from desalination plants: the distillation and the membrane process, to lessen the ecological impact [32].

The background of “Green Desalination” is based on the study “The state of desalination and brine production: A global outlook which was recently published in the journal *“Science of the Total Environment*”: Review: the state of desalination and brine production, and had a great impact on international media. The study was written by scientists from the Institute for Water, the Environment and Health from the United Nations University (UNU-INWEH), Wageningen University (Holland), and the Gwangju Institute of Science and Technology (South Korea) [33].

Study highlights

The need for unconventional water resources as the key to support the achievement of SDG 6.

Water production by desalination is 95.37 million m^3^/day.

Brine production and energy consumption are the key barriers to desalination expansion [33,34].

Four Middle Eastern countries produce about half of the world’s desalinated seawater. In order to reduce negative impacts on the environment and to reduce the water production costs, we have proposed innovative financial mechanisms and technological improvements (such as the development of commercial recovery and production systems for salts and metals). These solutions need to be affordable in order for them to be extended to low and middle income countries.

This research places emphasis on energy saving, cost saving, and environmental impact reduction, which follow the same direction as the “Mega-ton Water System” project as shown in Figure 7 [4,5,7].

The contents of the study conducted by the United Nations University were announced through two international TV media. The first one was Bloomberg News/9 January—“Saudi thirst for water is creating a toxic brine problem” and the second one was BBC News/14 January 2019—“Concerns over increase in toxic brine from desalination plants”. It remains unknown why both TV programs used the expression “toxic brine,” because the above study did not mention toxic brine. Saudi Water Conversion Corporation (SWCC) responded sensitively to the above journal and TV News, using advanced technology and science, emphasizing that water environment management is carried out at all stages of project development and implementation from planning to design, construction and operation. Furthermore, the Desalination Technology Research Institute (DTRI) of SWCC has announced that it is promoting a green desalination initiative, which has already produced tangible results [35,36]. The International Desalination Association (IDA) also responded to this journal and TV News, and in an effort to publicize the IDA’s response to the IDA Global Connections, conducted an opinion gathering survey on energy and environmental experts regarding the environmental impact of seawater desalination [36].

#### 3.1.2. History of Anti-Biofouling Trials

**Figure 18:**
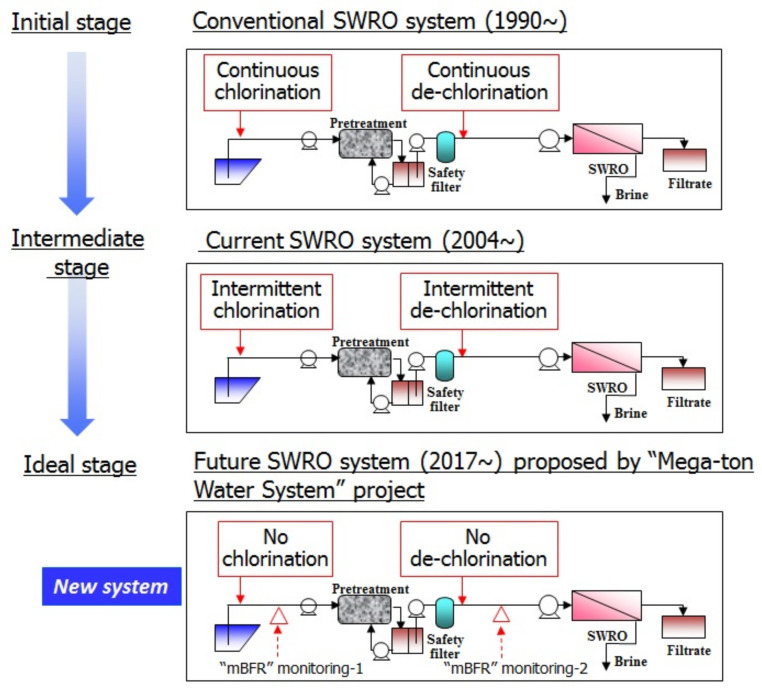
History of anti-biofouling trials and new system for future SWRO system [5,7,28].

As shown in Figure 18 [5,7,28], anti-biofouling trials for the SWRO system were conducted and the details are as follows:

(1) Initial stage: Conventional SWRO system since 1990.

Continuous chlorination and de-chlorination were conducted. As a result, almost all seawater desalination plants using polyamide membrane had heavy biofouling. However, this system is still being used in special cases, for example, when feeding seawater from power plants. In this system, sterilization by chlorination was considered to be common sense, but biofouling occurred on a frequent basis and constant chemical cleaning was performed. For example, two weeks of operation required one week of cleaning.

(2) Intermediate stage: current SWRO system (2004~)

Many global specialists on SWRO plant operation came together in Florida and San Diego in 2004, and discussed the heavy biofouling of SWRO plants using the conventional SWRO system.

As a solution, mild sterilization from continuous chlorination to intermittent chlorination has been adopted. However, biofouling has not been eliminated, and many specialists have doubts concerning the efficacy of chlorine sterilization.

(3) Ideal stage: Future SWRO system (2017~) presented by “Mega-ton Water System” project in Japan.

As the ideal stage, the future SWRO system (2017~) has been presented by the “Mega-ton Water System” project based on the data [5,7,28]. This is a paradigm shift in understanding that chlorine sterilization and SBS dosing trigger biofouling. Thus, the new system has no chlorination and no de-chlorination. As a result, biofouling can be controlled even in highly saline seawater such as that of the Gulf Sea. Recommendation: this method optimized the pretreatment conditions by monitoring the “mBFR”: membrane biofilm formation rate.

#### 3.1.3. Chlorine Sterilization Has No Effect on Marine Bacteria

**Figure 19:**
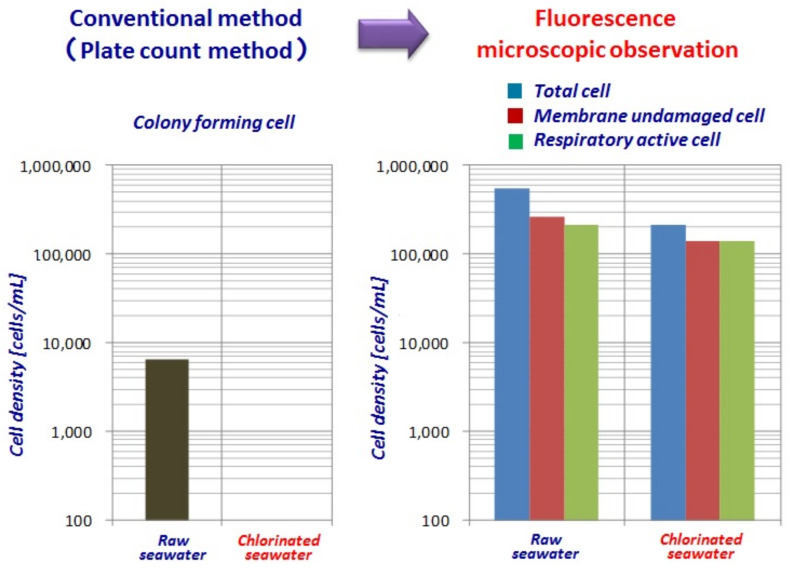
Chlorine sterilization of seawater has no effect [7,37,38,39].

Figure 19 shows the effect of chlorine sterilization on marine bacteria has been evaluated by comparing with the conventional method (Plate count method) and by fluorescence microscopic observation. As already reported by the authors as shown in Figure 19 [7,37,38,39], the plate count method can be overestimated and fluorescence microscopic observation shows that much higher numbers of marine bacteria survive after chlorination than expected. Chlorine sterilization of seawater is not only ineffective, but also counterproductive.

#### 3.1.4. “mBFR”: Membrane Biofilm Formation Rate

**Figure 20:**
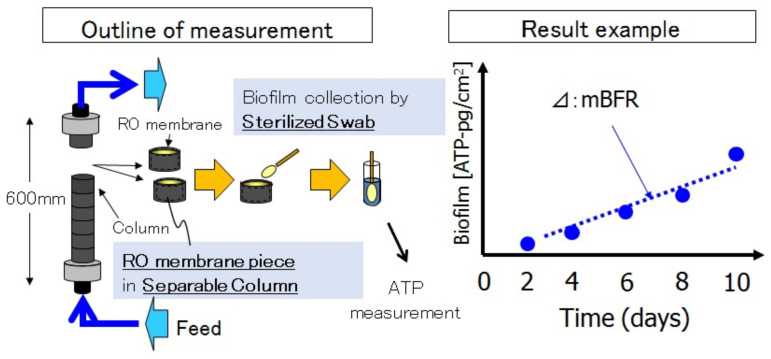
“mBFR”: Membrane biofilm formation rate [7,37,38].

The original “mBFR” measuring protocol was reported by the authors [7,37,38], after that, “mBFR”measurements much improved the accuracy and reliability of the biofilm formation evaluation as shown in Figure 20 by Ito et al. [7,37,38]. “mBFR” was developed by Toray as an indicator of the seawater RO feed water.

#### 3.1.5. Chlorine Sterilization and SBS Dosing Triggers Biofouling

**Figure 21:**
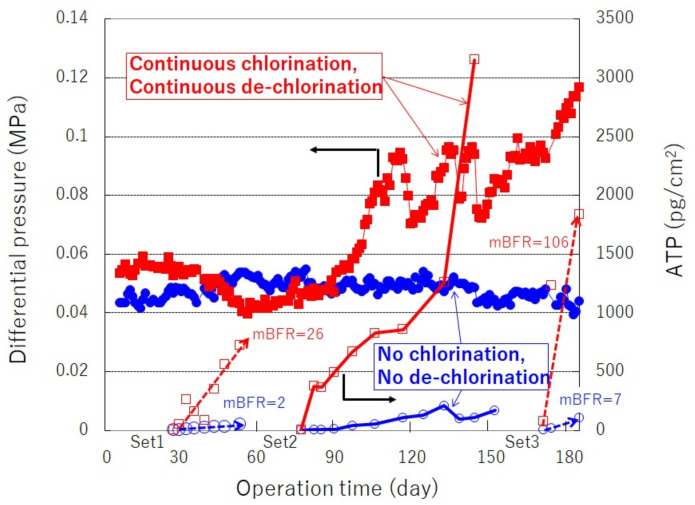
Chlorine sterilization and SBS dosing triggers biofouling [7,38].

In a real SWRO system as shown in Figure 18, shifted from an initial stage: conventional SWRO system that performs continuous chlorination and continuous de-chlorination to an ideal stage: Future SWRO system that performs no chlorination and no de-chlorination. These stages were compared in a pilot plant as shown in Figure 21 [7,38]. Figure 21 implies that no biofouling occurred in Future SWRO system compared to an initial stage. Chlorine sterilization and SBS dosing triggers heavy biofouling.

#### 3.1.6. Quantitative RO Chemical Cleaning Interval Due to Biofouling

**Figure 22:**
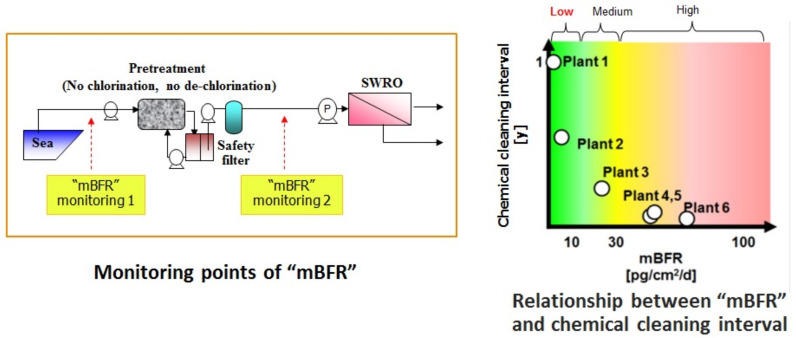
Quantitative RO chemical cleaning interval due to biofouling [7,37,38].

As shown in the left side of Figure 22, the points of “mBFR” are very important, in particular, the value of “mBFR” monitoring points as shown in Figure 22 [7,37,38]. The right side of Figure 22 shows the relationship between “mBFR” and the chemical cleaning interval. If the “mBFR” value at monitoring 2 is less than 10, the chemical cleaning interval will be necessary once or twice a year. Low “mBFR” is required for reliable operation of a SWRO plant with less chemical cleaning [7,38,39]. The chemical cleaning interval can be predicted based on “mBFR” of RO feed water [7,38,39]. The details were presented at the relevant international congress [7,38,39,40].

## 4. Future and Challenge of Membrane and Membrane Process

### 4.1. Prof. Rong Wong’s Review on SWRO Membrane Fabrication

Recently, Prof. Rong Wong et al. of Singapore Membrane Technology Center, Nangang Environment and Research Institute, Nanyang Technological University, reported in the title of “Seawater desalination by reverse osmosis: Current development and future challenges in membrane fabrication—A review” [41]. This report is well summarized based on the surveys conducted regarding patents and research papers presented by key manufacturers and universities on a global basis [41]. At the center of SWRO technology consists of thin film composite (TFC) membranes which not only promises a stable operation but also high separation performances.

The current state-of-the art SWRO membranes are the cross-linked fully aromatic polyamide composite-spirally wound membrane. Many approaches have been made to improve its performance by interfacial polymerization.

TFC PA membranes are currently the gold standard for SWRO desalination because of its ability to achieve much higher water permeability and salt rejection than asymmetric cellulose acetate-based membranes invented 60 years ago.

In the interfacial polymerization of optimization of selective layer as SWRO embedded membrane, the selection of monomers and fabrication condition, additives, and chemical compounds are recognized as being the main concerns. The main additives in the embedded membrane with cross-linked fully aromatic polyamide composite-spiral wound element are zeolite, CNT, silica and MOF (metal-organic frameworks), graphen oxide, and aquaporin. In particular, the author commented on researches in which the filler materials remained in the selective layer and contribute to the performance improvement not as the receipt of membrane preparation [41]. Likewise, Prof. Wong shows that the roadmap of the next generation SWRO membranes. Four Achilles are as shown in literature [41].

### 4.2. SWCC Future Plans for SWRO

**Figure 23:**
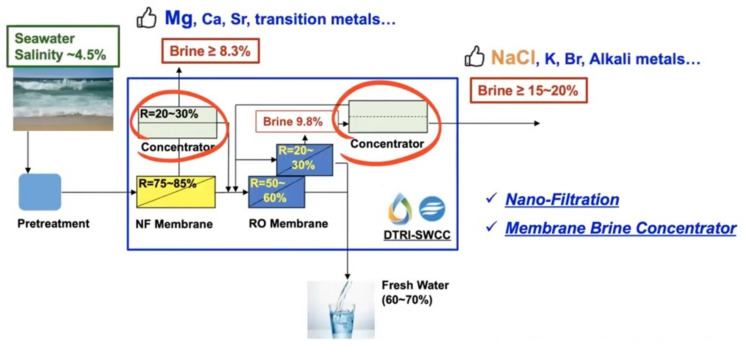
Dual Brine Concentrator [43].

The SWCC governor, HE. Abdullah Al-Abdul Kareem, presented the future SWCC plans with less CO_2_ emissions as shown in Table 3. This indicates that every item is quite important to achieve the next target of SWRO in Saudi Arabia [42] and also shows clean-cut quantitative targets not as qualitative. In particular, energy use for ZLD, their “new brine mining technology” has been introduced as one of the innovative technologies. The “Innovative Dual Brine Concentrator” is shown in Figure 23, which combines a NF membrane, RO membrane, and a membrane brine concentrator [43].

This technology enabled the cost effective mining of valuable minerals from the brine generated from desalination plants to produce commercial grade sodium chloride brine suitable for use by Chlor and Alkali industry [44]. If the innovative brine concentrator is developed, this market will expand greater than expected.

**Figure 24:**
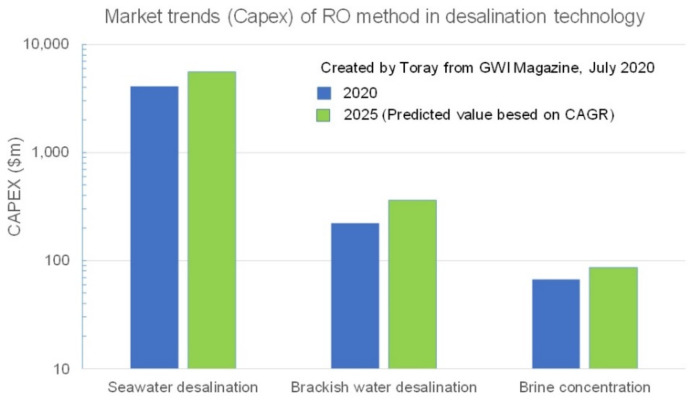
Market trends (Capex) of RO method in desalination technology [45].

Market trends (capex) of RO method in desalination technologies in 2020 and 2025 are shown in Figure 24 [45]. This figure illustrates that an RO membrane for seawater desalination is dominant, and then brackish water desalination, followed by a brine concentration, which is important to proceed to the next step. In this area, the thermal brine concentration is dominant now, but new technologies other than SWRO will be challenge this, or a hybrid system will become dominant in the near future [44].

## 5. Conclusions

(1) President Kennedy’s dream of obtaining fresh water from seawater has been realized as a great scientific achievement.

(2) As Tambo predicted, SWRO has become a major technology even in Middle Eastern countries.

(3) Current market status of SWRO plants has been changed dramatically.

Technology transition from distillation to membrane method.

From small plants to “Mega-size SWRO” plant.

New large plants excess of half mega-ton per day or mega-ton per day so-called “Mega-SWRO” are expected to be constructed over the next two to three years.

Affordable price of desalination water became as low as $0.53 to $0.28/m^3^ in the plant size of 545,000 to 909,200 m^3^/d.

(4) Since the invention of the Loeb-Sourirajan cellulose acetate membrane, 60 years have passed. As the commercial membrane, in particular, seawater desalination area, CTA-hollow fiber membrane and the cross-linked fully aromatic polyamide composite-spiral wound element are used, however, today, polyamide composite membrane has become the dominant commercial reverse osmosis membranes.

(5) Current status of SWRO technology.

Solar and wind power is a promising renewable resource to provide energy to operate the plants.

The innovative advanced low-pressure SWRO membrane and two-stage, high-recovery SWRO system contributes a 20% energy saving and a 30% energy saving has been confirmed by the SWRO-PRO hybrid system of the “Mega-ton Water System” project.

The hybrid system such as FO + SWRO or SWRO + PRO will be successful in the future.

## Figures and Tables

**Table 1:** Commercial SWRO membrane material and configuration.

Company	Membrane/ Element	Chemical Formula	Product in 2020	Data Resources
Dupont	B-10 -Asymmetric hollowfiber-	** 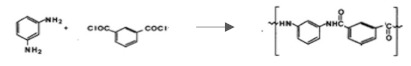 **	✖	1
	A-15 -Composite spiral-	** 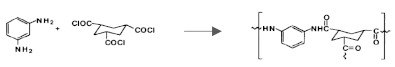 **	✖	2
FilmTec/ Dow/Dupont Water Solutions	FT-30 -Composite spiral-	** 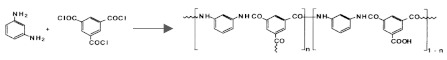 **	○	2
Toyobo	HOLLOSEP -Asymmetric hollowfiber-	** 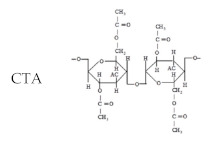 **	○	3
Toray	PEC-1000 -Composite spiral-	** 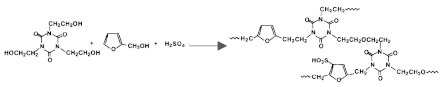 **	✖	2
	UTC-80 -Composite spiral-	** 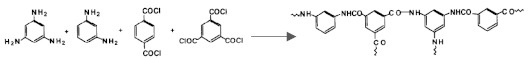 **	○	2
Nitto Hydranautics	-Composite spiral-	Similar to FT-30	○	-
LG Chem	-Composite spiral-	Similar to FT-30	○	-
**Data Resources:** 1. USP 3567632A 2. Peterson, R. J. Journal of Membrane Science,83(1993)81-150,Elsevier Science Publishers B. B:Amsterdam. 3. Takahito Nakao, Yuki Miura, Kenji Furuichi and Masahiro Yasukawa, *Membranes***2021**, *11*, 183. https://doi.org/10.3390/membranes11030183.				

Note: Product in 2020 ○: Available ✖: Unavailable.

**Table 2:** Products lineup for Toray’s SWRO [20].

	Specifications		
Product	TDS Rejection (%)	Water Productivity (GDP, (m^3^/day))	Boron Rejection (%)
TM820A	99.75	6000 (22.7)	93
TM820C	99.75	6500 (24.6)	93
TM820E	99.75	7500 (28.0)	91
TM820S	99.75	9000 (34.1)	90
TM820R	99.80	9400 (35.6)	95
TM820C	99.2	8800 (33.3)	94
TM820K	99.86	6400 (24.2)	96

**Table 3:** SWCC Future plans [42].

SWCC Future Plans
Exploration of renewable power alternative
Development of new generation of energy recovery devices, high pressure pumps and membranes
Energy use for desalination: less than 2.45 kWh/m^3^ as total energy
CO_2_ emission reduction for desalination plants: over 30%
Total energy use for new brine mining: less than 50% lower than the most advanced ZLD technologies at present

## Data Availability

Almost all data were prepared by the author and Toray Industries, Inc. The author used such data from his previous papers.

## Abbreviations:

Reverse osmosis (RO)	A method of separating water from dissolved salts by passing feed water through a semipermeable membrane at a pressure greater than the osmotic pressure caused by the dissolved salts.
Multi-stage flash evaporation (MSF)	A desalination process where a stream of brine flows through the bottom of chambers, or stages, each operating at a successively lower pressure and a proportions of it flashes into stream and is then condensed.
Multiple effect distillation (MED)	A thin film evaporation process where the vapor formed in a chamber, or effect con-denses in the next, providing a heat source for further evaporation.
Biofouling	Condition caused when bacteria growth forms a deposit on a filter membrane or heat transfer surface.
Pressure retarded osmosis (PRO)	PRO, proposed by Sidney Loeb, is a method of recovering the concentration difference (osmotic pressure) of energy using a separation membrane.
Seawater reverse osmosis (SWRO)	Desalination of seawater using reverse osmosis (RO) membrane.
High recovery SWRO system	High yield of permeate water from the feed seawater in SWRO system.
Green desalination	No chemical or less chemical system in SWRO system to save marine pollution and chemical cleaning of the plant.
“Mega-SWRO”	Large plants of the mega-ton per day scale (500,000 to 1,000,000 m^3^/day)
SWRO-PRO hybrid system	New energy recovery system from the brine of SWRO with the treated wastewater to use the salinity difference.
Brine concentration	SWRO needs the new technology for zero liquid discharge (ZLD) and precious material recovery from the brine SWRO plants.
“Mega-ton Water System” project	This project was funded by a grant from the Japanese Society of the Promotion of Science (JSPS).
GCC countries	UAE, Qatar, Kuwait, Saudi Arabia, Oman, and Bahrain
SEC	Specific energy consumption of the plant system (SEC: kWh/m^3^).
Renewable energy	Solar power and wind power energies.
BCS	Brine conversion system reducing the footprint, power, and cost of SWRO plants by high recovery operations.
